# Fecal Microbiota, Fecal Metabolome, and Colorectal Cancer Interrelations

**DOI:** 10.1371/journal.pone.0152126

**Published:** 2016-03-25

**Authors:** Rashmi Sinha, Jiyoung Ahn, Joshua N. Sampson, Jianxin Shi, Guoqin Yu, Xiaoqin Xiong, Richard B. Hayes, James J. Goedert

**Affiliations:** 1 Epidemiology and Biostatistics Program, Division of Cancer Epidemiology and Genetics, National Cancer Institute, 9609 Medical Center Drive, Bethesda, Maryland 20892–9704, United States of America; 2 Division of Epidemiology, Department of Population Health, New York University School of Medicine, 650 First Avenue, #518, New York, New York 10016, United States of America; 3 Information Management Services, 6110 Executive Boulevard, Rockville, Maryland 20852,United States of America; Singapore General Hospital, SINGAPORE

## Abstract

**Background and Aims:**

Investigation of microbe-metabolite relationships in the gut is needed to understand and potentially reduce colorectal cancer (CRC) risk.

**Methods:**

Microbiota and metabolomics profiling were performed on lyophilized feces from 42 CRC cases and 89 matched controls. Multivariable logistic regression was used to identify statistically independent associations with CRC. First principal coordinate-component pair (PCo1-PC1) and false discovery rate (0.05)-corrected P-values were calculated for 116,000 Pearson correlations between 530 metabolites and 220 microbes in a sex*case/control meta-analysis.

**Results:**

Overall microbe-metabolite PCo1-PC1 was more strongly correlated in cases than in controls (Rho 0.606 vs 0.201, P = 0.01). CRC was independently associated with lower levels of Clostridia, Lachnospiraceae, *p*-aminobenzoate and conjugated linoleate, and with higher levels of Fusobacterium, Porphyromonas, *p*-hydroxy-benzaldehyde, and palmitoyl-sphingomyelin. Through postulated effects on cell shedding (palmitoyl-sphingomyelin), inflammation (conjugated linoleate), and innate immunity (*p*-aminobenzoate), metabolites mediated the CRC association with Fusobacterium and Porphyromonas by 29% and 34%, respectively. Overall, palmitoyl-sphingomyelin correlated directly with abundances of Enterobacteriaceae (Gammaproteobacteria), three Actinobacteria and five Firmicutes. Only Parabacteroides correlated inversely with palmitoyl-sphingomyelin. Other lipids correlated inversely with Alcaligenaceae (Betaproteobacteria). Six Bonferroni-significant correlations were found, including low indolepropionate and threnoylvaline with Actinobacteria and high erythronate and an uncharacterized metabolite with Enterobacteriaceae.

**Conclusions:**

Feces from CRC cases had very strong microbe-metabolite correlations that were predominated by Enterobacteriaceae and Actinobacteria. Metabolites mediated a direct CRC association with Fusobacterium and Porphyromonas, but not an inverse association with Clostridia and Lachnospiraceae. This study identifies complex microbe-metabolite networks that may provide insights on neoplasia and targets for intervention.

## Introduction

The gut microbial population (microbiota) carries greater than 100-fold more genes than the human genome, through which it regulates numerous processes, such as energy harvesting, metabolism of dietary components, immunity, and activities of host or microbial derived chemicals.[1] Alteration or frank dysfunction of these processes is closely tied to inflammatory bowel disease, malnutrition and metabolic syndrome,[2–4] and it influences the risk for a wide range of diseases including colorectal cancer (CRC).[5–11] Whole-genome shotgun sequencing has provided insights on the metabolic potential of the gut microbiota, especially in studies that included transcriptomics.[1, 12–14] Targeted insights have come from studies of microbial consortia, dietary interventions, gnotobiotic mouse models, and transfer of fecal microbiota from diseased or healthy people.[3, 13, 15] Despite such progress, a comprehensive comparison of all detectable metabolites with all microbes in the distal human gut is lacking.

We have previously reported CRC associations with the fecal microbiota, specifically decreased relative abundance of Lachnospiraceae and other Clostridia and increased carriage of Fusobacterium, Atopobium, and Porphyromonas.[16] In the same population, CRC was associated with differences from the matched controls in levels of dozens of fecal metabolites.[17] Herein, we sought to uncover correlations between fecal microbes and metabolites and to identify statistically independent differences between CRC and matched controls.

## Materials and Methods

### Study participants and specimens

The study design has been described previously.[18, 19] Briefly, newly diagnosed cases with adenocarcinoma of the colon or rectum were recruited prior to surgery and treatment during 1985–1987.[18, 19] Controls were patients awaiting elective surgery for non-oncologic, non-gastrointestinal conditions at these hospitals during the same period. A median of 6 days (interquartile range, 3–13 days) prior to hospitalization and surgery, participants completed dietary and demographic questionnaires and provided two-day fecal samples that were frozen at home on dry ice and subsequently lyophilized. The two-day lyophilates were pooled, mixed and stored at -40°C. Participants provided written informed consent. The consent process and study procedures were reviewed and approved by an Institutional Review Board at the National Cancer Institute.[18, 19]

Of 69 cases and 114 controls in the original study,[18, 19] the case-control analysis included 48 cases and 102 controls for whom at least 100mg of lyophilized feces was available. Controls were frequency matched to cases by gender and body mass index (BMI). Microbiota and metabolomic analyses were conducted with these lyophilized fecal samples. As described previously,[16, 17] in both assays systems, the data were of excellent quality and highly reproducible. For the current analyses, there were 42 cases and 89 controls that had both metabolomics and microbiota data.

### Microbiota analyses

The details on the amplification, sequencing, classification and analysis of 16S rRNA genes are in Ahn et al.[16] Briefly, DNA was extracted using the Mobio PowerSoil DNA Isolation Kit (Carlsbad, CA). 16S rRNA amplicons covering variable regions V3 to V4 were generated, and the amplicons were sequenced with the 454 Roche FLX Titanium pyrosequencing system. Filtered sequences were binned into operational taxonomic units with 97% identity and aligned to fully-sequenced microbial genomes (IMG/GG Greengenes) using the QIIME pipeline.[20] The current analysis was restricted to the 220 microbes (across taxonomic levels, including 91 Firmicutes, 33 Bacteroidetes, 45 Proteobacteria, 11 Actinobacteria, 5 Fusobacteria, and 35 in other phyla) that were detected in at least 13 (10%) of the subjects.

### Metabolomics analyses

A range of small molecules (most <1000 Daltons) was detected in the lyophilized fecal specimens by high-performance liquid phase chromatography and gas chromatography coupled with tandem mass spectrometry (HPLC-GC/MS-MS, Metabolon, Inc., North Carolina, USA) as described previously.[21, 22] Briefly, non-targeted single methanol extraction was performed, followed by protein precipitation. Individual molecules and their relative levels were identified from the mass spectral peaks compared to a chemical reference library generated from 2,500 standards, based on mass spectral peaks, retention times, and mass-to-charge ratios. The molecules include, but are not limited to, amino acids, carbohydrates, fatty acids, androgens, and xenobiotics. Volatile molecules, such as short chain fatty acids, may be lost during lyophilization or extraction. However, such loss is generally equivalent across specimens, and lyophilization is optimal for fecal specimens to assure equal loading of dry weight. The current analysis was restricted to the 530 metabolites that were detected in at least 118 (90%) of the subjects.

### Statistical analyses

The overall objective was to identify covariation and possible interactions between fecal metabolites and fecal microbes, either associated with CRC or not. For the CRC association, we used unconditional logistic regression to calculate the odds ratio (OR) and 95% confidence interval (CI), with case status as the dependent variable and with each CRC-associated microbe as the primary independent variable;[16] age, sex, and BMI were included for empiric adjustment of potential confounding. Including race in the models had no substantive impact on the estimates. To each microbe model, metabolites were added in a forward stepwise logistic regression, and metabolites associated with CRC at P≤0.15 were retained. Change in OR with addition of metabolites was calculated as (OR_no metabs_−OR_metabs_) / (OR_no metabs_− 1). For standardized estimates, relative abundance of the microbes and natural-log levels of the metabolites were normalized to mean 0 and standard error 1. We also report the Pearson correlation coefficients between the metabolites and microbes that were associated with CRC.

For the global objective irrespective of CRC, we considered all 530 metabolites and 220 microbes, and used linear regression, stratified by sex and case status, to identify associations between metabolites and microbes. For each of the 530 x 220 regressions, we adjusted for age, race (White vs Other), BMI, and hospital. We illustrate the overall extent of associations by plotting the–log10(P-values) for each metabolite-microbe pairing in a “Manhattan” plot. We also calculated correlations of the top principal component (PC1) and principal coordinate (PCo1) of the metabolites and microbes, respectively. The principal components and principal coordinates were obtained from the residual matrix of linear regression models, adjusted for age, race, BMI, and hospital, for each metabolite or microbe, respectively. To compare the correlation ρ_1_, between PC1 and PCo1 in cases against the correlation ρ_2_ in controls, we applied Fisher’s Z-transformation Z(ρ) = 0.5ln((1+ρ/1-ρ)) to each correlation and then tested whether Z(ρ_1_)-Z(ρ_2_) was significantly different from 0. We assumed Z(ρ_1_)-Z(ρ_2_) was normally distributed with mean 0 and variance 1/(N_1_-3) + 1/(N_2_-3) under the null hypothesis where N_1_ and N_2_ are the number of cases and controls, respectively. The 2-sided P-value is 2*(1-P_norm_[(Z(ρ_1_)-Z(ρ_2_)]/sqrt(var)). Statistical analyses were performed in R version 3.1.0 (R Foundation for Statistical Computing, Vienna, Austria, http://www.R-project.org/).

## Results

Complete fecal microbiota and metabolome data were analyzed for 42 CRC cases and 89 age- and BMI-matched controls.[2] These 131 subjects had a mean age of 60 years (SD 13.2) and a mean BMI of 25.6 (SD 4.2); they were predominantly white and male (Table 1). Cases did not differ from controls on age, BMI, smoking or hospital, but a higher proportion of cases were African American and male (Table 1).

**Table 1:** Selected characteristics of colorectal cancer cases and controls with fecal metabolite data.

Characteristic	Colorectal cancer status	
	Cases (n = 42)	Controls (n = 89)
***Age***, mean (SD), years	63.4 (13.1)	58.4 (13.0)
***BMI***, mean (SD), kg/m2	25.8 (3.9)	25.5 (4.6)
***Sex***		
Male	59.5%	62.9%
Female	40.5%	37.1%
***Race***		
White	73.8%	85.4%
Black	23.8%	12.4%
Other	2.4%	2.2%
***Smoking***		
Never	54.8%	44.9%
Former	31.0%	46.1%
Current	11.9%	9.0%
Missing	2.4%	0.0
***Hospital***		
National Naval Medical Center	33.3%	41.6%
Walter Reed Army Medical Center	50.0%	37.1%
George Washington University Hospital	16.7%	21.3%
***Cancer stage***		
Non-invasive	21.4%	Not applicable
Invasive, no known metastases	42.9%	Not applicable
Metastatic	33.3%	Not applicable
Missing	2.4%	Not applicable

### Joint associations of fecal microbiota and fecal metabolites with CRC

In logistic regression models that included age, sex, and BMI, four microbes were significantly associated with CRC in separate models: Fusobacterium (OR 10.17, CI 2.95–35.0), Porphyromonas (OR 5.32, CI 1.76–16.05), Clostridia (OR 0.57, CI 0.38–0.85), and Lachnospiraceae (OR 0.61, CI 0.40–0.92). Table 2 presents these and the addition of fecal metabolites associated with CRC at a criterion of P≤0.15. In these models, the OR for CRC was approximately 2.8 with palmitoyl-sphingomyelin, 2.4 with *p*-hydroxy-benzaldehyde, 0.5 with *p*-aminobenzoate (PABA), and 0.5 with conjugated-linoleate-18-2N7 (CLA). Alpha tocopherol (OR 0.6) contributed to the Fusobacterium and Porphyromonas models, and mandelate (OR 1.6) contributed to the Clostridia and Lachnospiraceae models. With metabolites in the microbe models, the high OR of CRC with Fusobacterium was reduced by 29% (from 10.17 to 7.53), and the high OR with Porphyromonas was reduced by 34% (from 5.32 to 3.83). Attenuation of the low ORs with Clostridia and Lachnospiraceae was less marked (3.9% and 1.5%, respectively).

**Table 2:** Multivariable logistic regression models of fecal microbes and metabolites independently associated with colorectal cancer (CRC).*

Microbe alone	Microbe plus metabolites	Odds Ratio	95% Confidence Limits	
g_*Fusobacterium*	——————————————————-	10.17	2.95	35.00
	g_*Fusobacterium*	7.53	1.40	40.45
	Palmitoyl_Sphingomyelin	2.56	1.32	4.96
	*p*_Hydroxybenzaldhyde	2.75	1.34	5.63
	Conjugated linoleic acid (CLA)	0.47	0.24	0.93
	*p*_Aminobenzoate (PABA)	0.58	0.29	1.16
	Alpha_Tocopherol	0.57	0.29	1.12
g_*Porphyromonas*	——————————————————-	5.32	1.76	16.05
	g_*Porphyromonas*	3.83	1.03	14.22
	Palmitoyl_Sphingomyelin	3.28	1.70	6.33
	*p*_Hydroxybenzaldhyde	2.34	1.17	4.69
	Conjugated linoleic acid (CLA)	0.51	0.27	0.98
	*p*_Aminobenzoate (PABA)	0.56	0.29	1.11
	Alpha_Tocopherol	0.61	0.31	1.20
c_*Clostridia*	——————————————————-	0.57	0.38	0.85
	c_*Clostridia*	0.58	0.32	1.06
	Palmitoyl_Sphingomyelin	2.77	1.48	5.17
	*p*_Hydroxybenzaldhyde	2.40	1.19	4.86
	Conjugated linoleic acid (CLA)	0.59	0.31	1.11
	*p*_Aminobenzoate (PABA)	0.39	0.20	0.77
	Mandelate	1.64	0.93	2.90
f_*Lachnospiraceae*	-------------------------------------	0.61	0.40	0.92
	f_*Lachnospiraceae*	0.61	0.36	1.06
	Palmitoyl_Sphingomyelin	2.82	1.50	5.33
	*p*_Hydroxybenzaldhyde	2.24	1.09	4.61
	Conjugated linoleic acid (CLA)	0.56	0.30	1.05
	*p*_Aminobenzoate (PABA)	0.35	0.17	0.72
	Mandelate	1.60	0.92	2.78

* Age, body mass index (BMI), sex, and one microbe were included in each model. Metabolites associated with colorectal cancer (CRC) at a criterion of P≤0.15 were added to each microbe model. Microbe and metabolite levels were standardized to mean 0, standard error 1.

For further insight on the microbes and four metabolites that contributed to all of the logistic regression models, pairwise Pearson correlation coefficients were calculated by case-control status. In cases, strong correlations (|ρ|≥0.30) were found for three metabolite pairs: direct for linoleate-PABA, inverse for benzaldehyde-sphingomyelin and benzaldehyde-CLA (Table 3). Cases also had strong correlations of sphingomyelin with microbes, which were inverse with Clostridia and Lachnospiraceae and direct with Fusobacterium. Also in cases, Fusobacterium was directly correlated with Porphyromonas. Controls had few strong correlations: benzaldehyde-PABA (ρ = 0.30), Lachnospiraceae-PABA (ρ = -0.36), and Lachnospiraceae-Clostridia (ρ = 0.55). S1 Table presents, for cases and controls separately, the 20 metabolites that were most strongly correlated with each of the CRC-associated microbes.

**Table 3:** Pearson correlation coefficients for metabolites and microbes associated with colorectal cancer case status in multivariate analyses.*

**Metabolites and microbes**	**Correlation coefficients in cases**							
	Sphingomyelin	Benzaldehyde	Linoleate	PABA	Clostridia	Lachnospiraceae	Fusobacterium	Porphyromonas
Sphingomyelin	1	***-0*.*36***	0.03	0.08	***-0*.*30***	***-0*.*33***	***0*.*44***	0.10
Benzaldehyde		1	***-0*.*45***	-0.14	0.14	0.09	*-0*.*24*	0.08
Linoleate			1	***0*.*44***	0.11	0.11	0.06	0.02
PABA				1	0.13	-0.11	-0.15	0.16
Clostridia					1	***0*.*70***	*-0*.*20*	0.17
Lachnospiraceae						1	*-0*.*27*	-0.09
Fusobacterium							1	***0*.*42***
Porphyromonas								1
**Metabolites**	**Correlation coefficients in controls**							
**and microbes**	Sphingomyelin	Benzaldehyde	Linoleate	PABA	Clostridia	Lachnospiraceae	Fusobacterium	Porphyromonas
Sphingomyelin	1	-0.02	0	-0.15	-0.12	-0.14	0.12	0.05
Benzaldehyde		1	-0.16	***0*.*30***	0.04	-0.08	-0.03	0.04
Linoleate			1	-0.02	-0.02	-0.08	0.05	-0.04
PABA				1	-0.12	***-0*.*36***	0.02	-0.07
Clostridia					1	***0*.*55***	0.07	-0.04
Lachnospiraceae						1	-0.16	-0.12
Fusobacterium							1	0.13
Porphyromonas								1

* Relative abundance of class-level Clostridia and Lachnospiraceae; carriage (detection/non-detection) of genus-level Fusobacterium and Porphyromonas. PABA indicates *p*-aminobenzoate. ***Bold italic*** P<0.05. *Italic* P<0.10.

### Associations of the fecal microbiota with fecal metabolites

**Fig 1:**
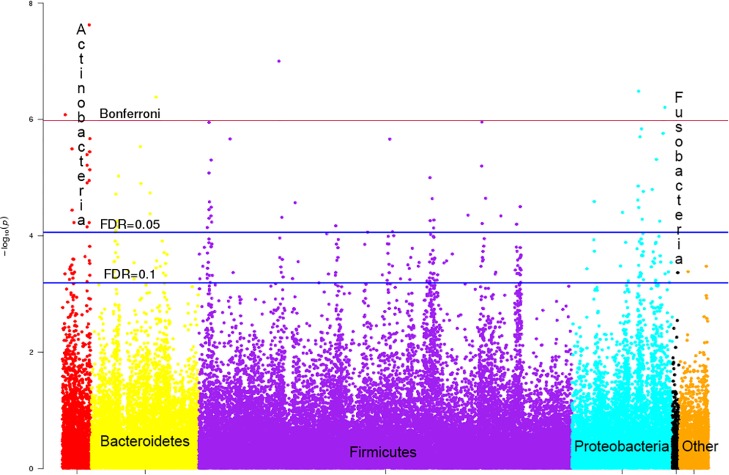
Observed inverse (-log10) meta-analyzed P-values for associations between 530 fecal metabolites and 220 fecal microbes. Microbes are color coded by phylum (Actinobacteria, red; Bacteroidetes, yellow; Firmicutes, purple; Proteobacteria, cyan; Fusobacteria, black; other phyla, orange) and sorted by genus. Bonferroni and false discovery rate (FDR) 0.05 and 0.1 threshold lines are presented.

To further explore the association between the microbiota and metabolites, we conducted the principal component/coordinate analysis of all 530 metabolites and 220 microbes. We found that the correlation between metabolite PC1 and microbial PCo1 was much stronger in CRC cases than in controls (Rho 0.606 vs 0.201, P = 0.01). For an overall view, we used a 4-group meta-analysis (sex*case/control), further adjusted for age, BMI, race, and enrollment hospital. Fig 1 presents all 116,600 (530*220) meta-analyzed P-vales by microbial phylum. At the FDR = 0.1 threshold, there were 263 significant metabolite correlations, including 32 (12%) with Actinobacteria, 54 (20%) with Proteobacteria, 141 (54%) with Firmicutes, 33 (13%) with Bacteroidetes, 1 (0.3%) with Fusobacteria, and 2 (0.7%) with microbes in other phyla. At the FDR = 0.05 threshold, there were 72 significant metabolite correlations, including 14 (19%) with Actinobacteria, 15 (21%) with Proteobacteria, 31 (43%) with Firmicutes, 12 (17%) with Bacteroidetes, and none with Fusobacteria or microbes in other phyla. S5 Table presents exploratory associations of CRC with these 72 FDR = 0.05-significantly correlated microbe-metabolite pairs. In these 72 logistic regression models, CRC had a nominal direct association with 2-aminobutyrate (OR 1.60, CI 1.07–2.39) and g_Arcobacter (phylum Proteobacteria, OR 1.94, CI 1.17–3.22), and it had a nominal inverse association with unknown metabolite X_17626 (OR 0.59, CI 0.39–0.91) and g_Ruminococcus (phylum Firmicutes, OR 0.59, CI 0.35–0.99).

The 4 Bonferroni-significant inverse correlations were indolepropionate with Actinomyces (Actinobacteria), threnoylvaline with Bifidobacterium (Actinobacteria), alanylalanine with Catabacteriaceae (Firmicutes), and 2-aminobutyrate with Butyricimonas (Bacteroidetes); the 2 Bonferroni-significant direct correlations were erythronate with Enterobacteriaceae (Proteobacteria) and an uncharacterized metabolite with Klebsiella (Proteobacteria).

**Fig 2:**
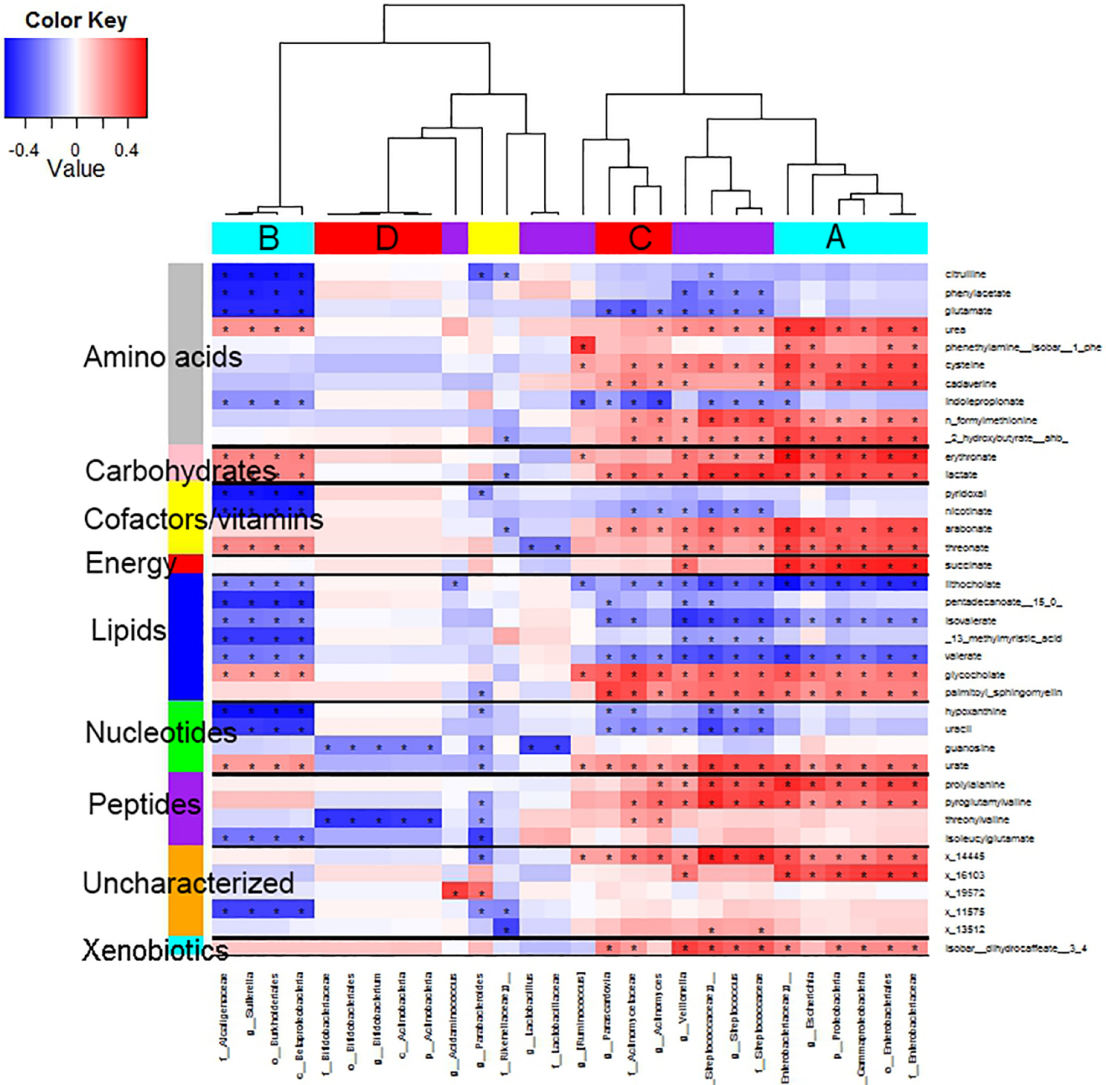
Heat map of the 100 strongest values of Pearson correlation coefficients of the residuals of all 530*220 fecal metabolite-microbe pairs. Asterisk (*) indicates correlation significant at false discovery rate (FDR) 0.2. Bars at the top are color coded by phylum, as in Fig 1 (Proteobacteria, cyan; Actinobacteria, red; Firmicutes, purple; Bacteroidetes, yellow). Clusters are arbitrarily labeled A, B, C, and D. Bars on the left are color coded by metabolite pathway, as indicated.

Two clusters of Proteobacteria had distinct metabolite correlations (Fig 2, cyan bars A and B). Cluster A (Gammaproteobacteria, particularly Enterobacteriaceae) had inverse correlations with three lipids (lithocholate, isovalerate, and valerate), and this cluster had strong direct correlations with six amino acids, two carbohydrates (erythronate and lactate), two cofactors/vitamins (arabonate and threonate), one energy (succinate), two lipids (glycocholate and palmitoyl-sphingomyelin), one nucleotide (urate), two peptides, two uncharacterized metabolites, and a xenobiotic (dihydrocaffeate). Cluster B (Betaproteobacteria, particularly Alcaligenaceae) had direct correlations with the same carbohydrates, erythronate and lactate; but most of the other correlations differed for clusters A and B (Enterobacteriaceae and Alcaligenaceae). Cluster C comprised three Actinobacteria and five Firmicutes; and it differed from cluster A predominantly by inverse correlations with three amino acids, one cofactor/vitamin, and two nucleotides. Cluster D included five Actinobacteria (particularly Bifidobacteriaceae) that were inversely correlated with guanosine and threonylvaline. Parabacteroides, the only microbe inversely correlated with palmitoyl-sphingomyelin, was also inversely correlated with three dipeptides and three nucleotides.

## Discussion

By comparing a comprehensive profile of the microbiota to a comprehensive panel of metabolites in the same specimens, the current study revealed microbe-metabolite correlations in human feces. It also revealed statistically independent microbe-metabolite differences between CRC cases and matched controls. These findings complement the metagenomic and animal-model studies that have identified characteristics of the distal human gut microbiota that are associated with CRC, inflammatory bowel disease, metabolic syndrome, obesity or malnutrition.[1–4, 11, 13, 14, 16] Overall, in 131 individuals we found 72 correlations between fecal metabolites and microbes that were significant at the FDR 0.05 level, of which six were significant at the Bonferroni level. The highly diverse Firmicutes phylum had 43% of the FDR-significant correlations, whereas the highly conserved Fusobacteria and other rare phyla had none. Microbe-metabolite correlations were significantly stronger in CRC cases than in controls. Directly comparing cases to controls, CRC was associated with significantly lower levels of Clostridia, Lachnospiraceae, PABA and CLA, and with higher levels of Fusobacterium, Porphyromonas, palmitoyl-sphingomyelin and *p*-hydroxy-benzaldehyde.

Our Bonferroni-significant microbe-metabolite pairs should be noted. Butyricimonas, a butyrate-producing genus in the family Porphyromonadaceae (Bacteroidetes), was inversely correlated with 2-aminobutyrate and apparently caused septic shock in a recently reported CRC patient.[23] Four other septic patients yielded the discovery of *Catabacter hongkongensis*,[24, 25] which is the sole member of the new Catabacteriaceae (Firmicutes) that we found to be inversely correlated with a fecal dipeptide. Proteobacteria and Actinobacteria were correlated with several metabolites. Enterobacteriaceae (Gammaproteobacteria), which includes Klebsiella, Escherichia, Shigella, Salmonella, Serratia, and other pathogens, were directly correlated with an uncharacterized metabolite and with erythronate, a product of hyaluronic acid metabolism and oxidative stress.[26, 27] Of the Actinobacteria, some Streptomyces species produce a wide range of commonly used antimicrobial medications and other metabolites;[28] and Actinomyces had a Bonferroni-significant inverse association with indolepropionate in our study.

Of 11 fecal metabolites associated with CRC in univariate analysis,[17] only four were independently associated with the malignancy when adjusted for each other and for a CRC-associated microbe (Table 2). This reflects, at least in part, the correlations of several metabolites with each other (Table 3) and perhaps shared pathways.[17] Nonetheless, the CRC associations with these four metabolites (PABA, CLA, palmitoyl-sphingomyelin, and *p*-hydroxy-benzaldehyde) were only modestly attenuated when they were mutually adjusted for each other. Similarly, these metabolites minimally attenuated the CRC association with two low-risk microbes (Clostridia and Lachnospiraceae). In contrast, CRC association with the high-risk microbes (Fusobacterium and Porphyromonas) was attenuated 40–53% by the metabolites, suggesting that these metabolites mediate, in part, the association of Fusobacterium and Porphyromonas with CRC.

As reviewed elsewhere,[29] the microbiota produces thousands of chemically diverse molecules that potentially affect human health. How such microbial metabolites, including those in Table 2, affect or mark CRC risk is unknown. Possible mechanisms include shedding of cell membranes due to microbial invasion;[30–32] modulation of bacterial replication, inflammation, and cancer;[33–37] and synthesis of PABA and antibiotic precursors.[38–40]

This study had important limitations. First, the representativeness of the metabolites detected in our 20 year-old specimens is unknown, although they were stored in a lyophilized state at or below -40°C. Second, our study did not formally dissect the interactions of the highlighted metabolites and microbes. This might be accomplished by study of systematically constructed microbial consortia.[15] Third, while the microbe-metabolite correlations considered the multiplicity of comparisons, the associations with CRC did not. Despite this, both the microbe-metabolite and the CRC associations present hypotheses for independent or joint effects that can be examined in future studies. Fourth, we lacked an additional set of specimens for external validation. However, by focusing on a fixed set of the top metabolites, we obtained an estimate of the upper bound of the effect of the metabolites on each of the CRC-associated bacteria. Fourth, although our study considered 530 small molecules, it did not employ state-of-the-art holistic platforms that detect up to 10-fold more fecal metabolites,[41, 42] nor did it specifically probe immunologic and inflammatory pathways that are centrally involved in CRC pathogenesis.[10, 43, 44] Finally, we have not identified functions of the fecal microbes that we detected. Previously, we noted that the activities of two important enzymes in feces, β-glucuronidase and β-glucosidase, were directly correlated with microbiota alpha diversity and abundance of Clostridia, and inversely correlated with abundances of Streptococcus and Alistipes.[45] Others have shown that the microbiota of specific pathogen-free mice can generate anti-inflammatory regulatory T cells, which moderate systemic immunity, through the production of butyrate.[46]

In summary, this study uncovered a complex network of microbes and molecules in human feces. In this network, CRC cases had strong microbe-metabolite correlations that were predominated by Proteobacteria and Actinobacteria. To obtain insights on disease and to identify targets for intervention, functional studies will be needed. Ultimately, innovative prospective human studies, including clinical trials, will be required.[15, 47]

## Supporting Information

S1 Table Metabolites most strongly correlated with colorectal cancer-associated taxa, by case-control status. (XLSX)

S2 Table Standardized levels of all metabolites among study participants. (CSV)

S3 Table Relative abundance of all microbial taxa among study participants. (CSV)

S4 Table Colorectal cancer (CRC) case-control status, covariates, and logistic regression beta values for associations of candidate metabolites and microbial taxa with CRC. (CSV)

S5 Table Logistic regression association with colorectal cancer (CRC) for the 72 metabolite-microbial taxon pairs that were correlated with each other at false discovery rate (FDR) 0.05. (XLSX)

## Abbreviations

BMI: body mass index
CRC: colorectal cancer
CI: confidence interval
FDR: false discovery rate
OR: odds ratio
PABA: *p*-aminobenzoate
PC: principal component
PCo: principal coordinate
SD: standard deviation
